# Reproducing the hierarchy of disorder for *Morpho*-inspired, broad-angle color reflection

**DOI:** 10.1038/srep46023

**Published:** 2017-04-07

**Authors:** Bokwang Song, Villads Egede Johansen, Ole Sigmund, Jung H. Shin

**Affiliations:** 1 Department of Physics, KAIST, 335 Gwahangno, Yuseong-Gu, Daejeon, Rep. of Korea; 2 Department of Chemistry, University of Cambridge, Lensfield Road, CB2 1EW, Cambridge, United Kingdom; 3 Department of Mechanical Engineering, Solid Mechanics, Technical University of Denmark, Bld. 404, DK-2800 Kgs. Lyngby, Denmark; 4 Graduate School of Nanoscience and Technology KAIST, 335 Gwahangno, Yuseong-Gu, Daejeon, Rep. of Korea

## Abstract

The scales of *Morpho* butterflies are covered with intricate, hierarchical ridge structures that produce a bright, blue reflection that remains stable across wide viewing angles. This effect has been researched extensively, and much understanding has been achieved using modeling that has focused on the positional disorder among the identical, multilayered ridges as the critical factor for producing angular independent color. Realizing such positional disorder of identical nanostructures is difficult, which in turn has limited experimental verification of different physical mechanisms that have been proposed. In this paper, we suggest an alternative model of *inter-structural disorder* that can achieve the same broad-angle color reflection, and is applicable to wafer-scale fabrication using conventional thin film technologies. Fabrication of a thin film that produces pure, stable blue across a viewing angle of more than 120 ° is demonstrated, together with a robust, conformal color coating.

Intricate structures^1^,^2^,^3^,^4^,^5^ create structural colors that can remain brilliant after millions of years of fossilization^6^. One of the most well-known examples is the butterflies of genus *Morpho*^7^ whose bright, blue wings grace many famous collections, and are reported to be visible even from low-flying aircrafts^8^. Yet, while structural in nature, their colors are also famous for remaining relatively stable over a wide range of viewing angles, contrary to the very definition of iridescence^9^. This seeming paradox has attracted a great deal of attention, and by now, there is a general agreement that the color is due to: multilayered ridge structure that provides strong, selective reflection in the blue^10^,^11^; narrow, sub-wavelength width of the ridges that provide broad-angle diffraction^12^,^13^; and formation of a close-packed array of such narrow, discrete multilayered ridges on the wing scales to provide a high reflectivity^13^. When the ridges are arranged periodically, however, they take the form of a grating, with narrow, strong grating peaks and angle-dependent color. In the case of *Morpho* butterflies, most investigations have identified the sub-wavelength scale positional disorder among the multilayered ridges as the critical factor that prevents a diffraction grating appearance, in turn providing blue reflection that is both bright and broad^9^,^14^,^15^,^16^,^17^,^18^. Thus, much of the research has been focused on developing methods of analyzing and fabricating model structures that consist of arrays of identical multilayer reflectors with varying degrees of positional disorder^13^,^18^,^19^,^20^,^21^. While many methods of analysis have been developed successfully^9^,^15^,^16^,^17^,^18^, actually fabricating the corresponding model structure of an array of identical ridges with positional disorder has been challenging. Consequently, reported *Morpho*-mimetic arrays were either periodic with strong, angle-dependent diffraction peaks^22^,^23^,^24^, or planar, 2D structures without distinctive ridged structure^13^,^19^,^25^. This not only led to a dearth of quantitative, experimental verification of many conflicting theories regarding *Morpho* butterflies, but also prevented developing a practical application for *Morpho*-mimetic structure despite its advantage in applications such as displays^26^,^27^, sensing^28^,^29^,^30^,^31^, and energy conversions^32^.

In this paper, we propose introducing *inter-structural disorder* (defined to be random variations in the internal structure of reflecting ridges), as an alternative that works just as well in generating the incoherence among the reflection from ridges, which is fundamental for reducing the angle-dependence of color^14^,^18^,^33^. More importantly, introducing such *inter-structural disorder* is a bulk-focused method since the disorder lies in the difference between the internal structure of each unit and not the individual shape, which in turn allows to focus on controlling the statistical parameters of disorder, not the individual shape. Based on these findings, we demonstrate fabrication of a dense array of narrow, multilayered ridges with *inter-structural disorder*. Blue color that remains stable across a viewing angle of more than 120 ° is achieved from a planar film that is only ~2 μm thick. Consequently, the film enables realization of ultra-thin coatings that conformally can coat fine-featured surfaces, while being robust enough to survive repeated soaking and folding in liquid nitrogen without visible damage.

## Results

### Inter-structural disorder

**Figure 1:**
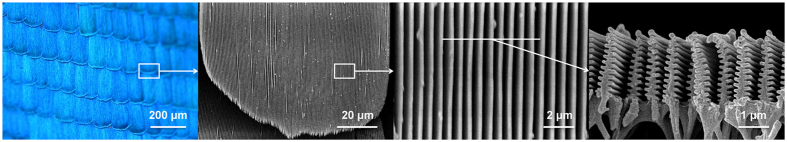
Images of *Morpho Rhetenor*. An optical microscope image (far left) and three scanning electron microscope (SEM) images of a *Morpho Rhetenor* butterfly, showing the structure in increasing detail.

We start by investigating the ridge structure of a *Morpho Rhetenor* butterfly. A series of images at different scales is shown in Fig. 1. Several points are noteworthy. First, the ridges are not identical. Second, as the ridges are several micrometers tall, but with sub-micrometer widths, and taper to a point at the top, a well-defined “layer” is difficult to define. These observations clearly question the widely used model of an array of identical multilayered ridges.

**Figure 2:**
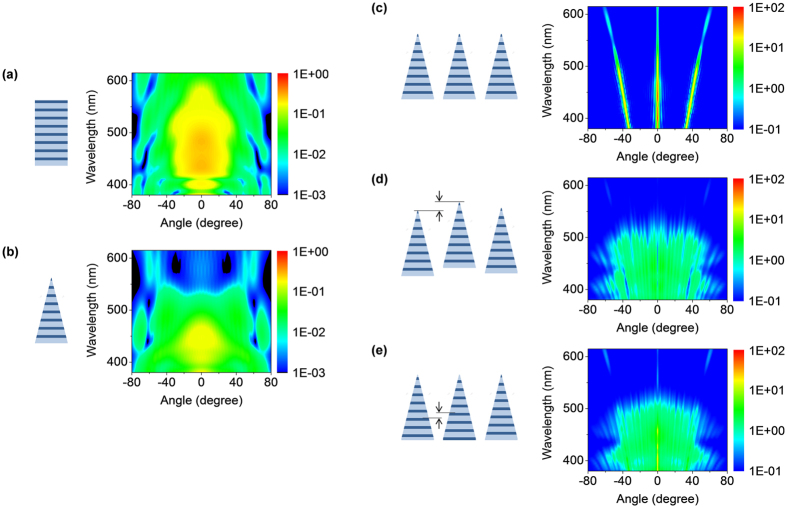
Structural modelling. (**a,b**) Calculated unit responses of (**a**) a rectangular ridge with regular layers, (**b**) a tapered, triangular ridge with regular layers. The base width of the ridges is 500 nm. (**c-e**) Calculated reflection spectra of (**c**) a periodic array of tapered ridges, (**d**) a periodic array whose ridges are displaced randomly in the vertical direction between ±110 nm, and (**e**) a periodic array of ridges whose internal multilayers are displaced randomly in the vertical direction between ±110 nm while keeping the external shape and the layer periodicity the same. All calculations are performed under normal incident light conditions. Note that all graphs are plotted in log scale with arbitrary units. In all cases, a schematic of the corresponding structure is given.

Therefore, in order to develop a *Morpho*-inspired structure that can provide broad-angle blue reflection, we now treat the entire ridge as a single, fundamental unit of reflection, and investigate its reflection spectrum using numerical simulations. As a model system, we choose SiO_2_/TiO_2_ multilayers. SiO_2_/TiO_2_ multilayer systems have been used previously in planar-type *Morpho*-inspired reflectors^13^,^34^,^35^, and have a large index contrast such that only 8 pairs are needed to achieve full reflection. Based on previous research, the SiO_2_ and TiO_2_ layer thicknesses were kept at 95 nm and 45 nm, respectively, to achieve a reflection maximum in the blue regime^36^. Figure 2a shows the calculated reflection spectrum of a rectangular ridge with flat, regular layers under normal illumination. The angular response is very broad due to diffraction by the narrow ridge. The maximum reflection occurs near 450 nm, as expected. For comparison, we also calculate the reflection spectrum of a triangular ridge with a base width of 500 nm, as a simplified model of the tapered ridge of the *Morpho* butterfly shown in Fig. 1. The result is shown in Fig. 2b. We find that the reflectance of the longer wavelength region is highly suppressed resulting in a shift towards blue of its hue^33^. Detailed numerical investigations indicate that a triangular ridge tends to show such a suppression, but the spectral details depend on the actual ridge shape (See Supplementary Information, Fig. S1 for more details).

However, using a single ridge is unrealistic for both *Morpho* butterflies and practical applications. As already mentioned above, achieving a high overall reflectivity requires packing the ridges in a dense array. When the array is periodic, however, the overall reflection is dominated by sharp grating peaks that are strongly angle-dependent, as shown in Fig. 2c, which illustrates the calculated reflection spectrum from a periodic array of such tapered identical ridges. The key solution, that has been the focus of models so far for removing such grating peaks to achieve high, broad-angle reflection, is positional disorder. As shown in Fig. 2d, introducing positional disorder by randomly displacing the ridges vertically between ±110 nm prevents grating formation, and results in broad-angle blue reflections. So far, such an array of ridges with a similar continuous positional disorder has been challenging to fabricate with conventional thin-film technologies. The main role of positional disorder is to introduce random phase differences in the reflections from each ridge to remove their coherence, with height disorder having the dominating effect^13^,^17^,^19^. We propose to achieve the same effect by introducing *inter-structural disorder* through randomly varying the relative positions of the internal multilayers while keeping the external shape of the ridges the same. The calculated reflection spectrum of a hypothetical array with *inter-structural disorder* is shown in Fig. 2e. We find that *inter-structural disorder* suppresses grating modes to such an extent where they are hardly visible on a surface.

Interestingly, we find that for more complex structures with *inter-structural disorder*, the scattering from each individual unit may not represent the scattering observed from an ensemble of units or a simple averaging thereof. The difference between analyzing as an ensemble or simple averaging is discussed in more detail in Figure S2(g). Furthermore, including stochastic parameters in the analysis process allows inclusion of fabrication tolerances in the design phase. This can be actively used in the design phase instead of spending effort on minimizing nanoscale tolerances to obtain an ideal replication of a model. For bulk processes, we believe this is a more viable approach.

### Fabrication of Morpho-inspired structure

**Figure 3:**
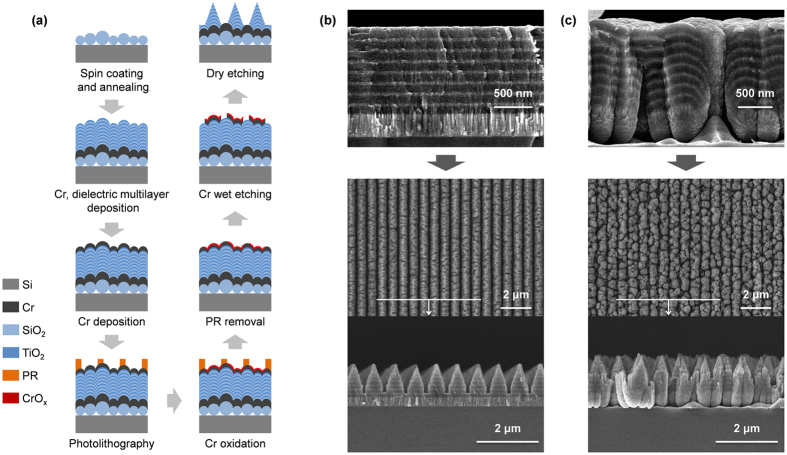
Structural analysis. (**a**) A schematic description of fabrication process for *Morpho*-inspired structure with *inter-structural disorder*. (**b**) SEM images of the regular multilayer before ridge formation (top) and after ridge formation. (**c**) Same as (**b**), but with irregular layers.

To fabricate a ridge array with *inter-structural disorder*, we utilize the randomly sized silica ball substrate that we have reported before as a basis^34^. As shown schematically in Fig. 3a, a multilayer film with 8 pairs of SiO_2_ and TiO_2_ layers with thicknesses of 95 nm and 45 nm, respectively, was deposited on a monolayer of randomly sized silica microspheres with diameters ranging from 250 to 440 nm preserving the random height variations of the substrate such that the layers are irregular. Subsequent lithography and etching created a dense array of ridges with a periodicity of 700 nm. For comparison, a similar array without *inter-structural disorder* was fabricated by depositing the same multilayer structure on a Si wafer such that the layers are regular (see Methods section for more details). Figure 3b,c show the SEM images of the fabricated structures, together with their schematic descriptions. We find that both structures have the same external tapered shape and periodicity. The critical difference lies in the internal structures, however. The ridges formed on the Si wafer are all identical. In contrast, each of the ridges formed on the randomly sized silica ball substrate are distinct from each other due to the irregularity of the original layers.

### Optical analysis

**Figure 4:**
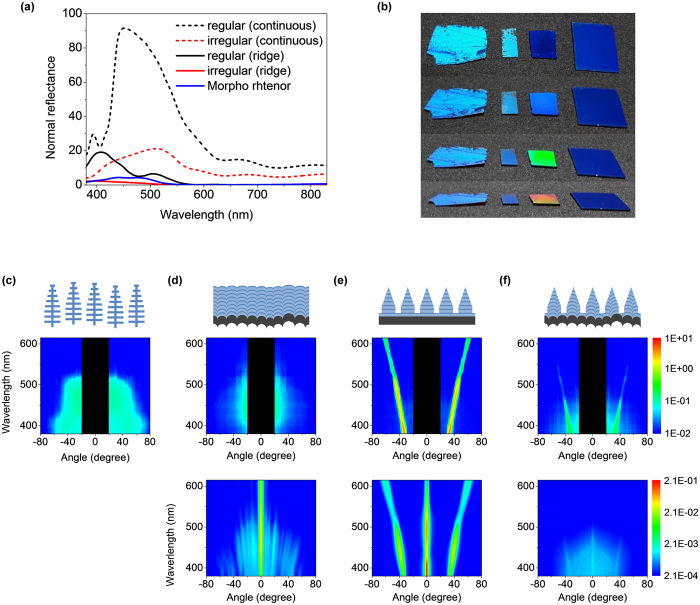
Optical analysis. (**a**) Normal reflectance spectra of the continuous (before ridge formation) film with regular and irregular layers, and the ridge structures with regular and irregular layers, and *Morpho Rhetenor*. (**b**) Optical images that compare an actual *Morpho Rhetenor* wing with different films fabricated in this paper under normal illumination. From left to right: the wing of *Morpho Rhetenor*, a continuous structure with irregular layer (as shown in the top of Fig. 3c), a ridge structure with regular layers (as shown in Fig. 3b), and a ridge structure with irregular layers (as shown in Fig. 3c). The viewing angles are, from the top, approximately 10, 40, 50, and 60 degrees. The direction of a ridge is aligned perpendicular with rotational plane of viewing angle. (**c**) Experimentally measured reflection spectra of *Morpho Rhetenor* under normal incident light conditions. A sketch of the corresponding structure is given on top. The experimental data are given in absolute reflectance values calibrated by an Al mirror. (**d**) Experimentally measured (middle) and calculated (bottom) reflection spectra of a fabricated, continuous structure with irregular layers under normal incident light conditions. The simulated values are normalized according to ref. 38 and multiplied by cosine to the reflection angle in order to convert to irradiance. Due to finite detector size, the relation between measurement and simulation are relative. (**e**) Same as (**d**), but with a fabricated, tapered ridge structure with regular layers. (**f**) Same as (**d**), but with a fabricated, tapered ridge structure with irregular layers. Simulated data has been Gaussian blurred as a simple way of incorporating the effect of the finite-sized detector in the measurement setup. Note that all graphs are plotted in log scale.

The normal reflectance spectra of the films are shown in Fig. 4a. We find that formation of the tapered ridge highly suppresses the normal reflection, especially in the longer wavelength region. When the viewing angle is changed, however, the colors diverge dramatically, as is shown in Fig. 4b. The color of the ridged film with regular layers changes all over the visible spectrum as expected. In contrast, the color of the ridged film with *inter-structural disorder* remains blue at all viewing angles. For a more quantitative analysis, the angle-dependent reflection spectra for *Morpho Rhetenor* and the fabricated structures are shown in Fig. 4c–f respectively. Also shown for comparison are the theoretical reflection spectra of the fabricated structures calculated using the measured irregular shapes of layers and the actual shape of the ridges obtained from SEM images (See Supplementary Information, Fig. S2 for more detailed information on simulation structures). In both cases, we observe a good agreement between calculated and experimental results. Without *inter-structural disorder*, the spectrum is dominated by sharp peaks confirming that the array indeed is a grating, with a maximum reflection peak near 400 nm and a strong suppression of all reflection in the red (see Supplementary Information, Fig. S3 for the calculated normal reflectance spectra). With *inter-structural disorder*, we observe a nearly 100-fold reduction in the intensity of grating peaks, and the generation of broad, uniform reflection in the blue, confirming that the *inter-structural disorder* has reduced the coherence of reflection from the multilayered ridges to suppress the grating effect, despite the identical external shape of the ridges. Such broad-angle reflection is maintained for an oblique incidence angle as well (For the measured and calculated reflection spectra for a 45 ° incident angle and angle-resolved spectra of selected wavelengths, see Supplementary Information, Fig. S4).

Despite the strong suppression, grating peaks still remain for the tapered ridge structure with *inter-structural disorder*, indicating that the fabricated disorder is not sufficient to fully generate a broad-angle blue reflection. This insufficient suppression also involves somewhat low brightness for non-diffracting viewing directions. Regarding fabrication, the reason for lack of disorder is the lack of directionality of the deposition process, which cannot preserve the random height variations of the substrate perfectly. After the deposition process, the standard deviation of vertical disorder at the surface is reduced to 20 nm, while that of the original substrate is 46 nm according to AFM and SEM measurement data (see Supplementary Information, Fig. S2 for the data measured by AFM and SEM). Therefore, improving the directionality can be one of the solutions for increasing disorder (see Supplementary Information, Fig. S5 for the analysis of directionality effect). Increasing microsphere size distribution can be another solution. Adding positional disorder^17^,^18^ could also suppress the remaining grating response, but is not straight-forward using the current fabrication process. For achieving longer wavelength colors like green or red, upscaling the disorder is indispensable (see Supplementary Information, Fig. S6 for details on creating a red sample).

It is important to point out that the achieved broad-angle reflection is not due to the irregular, non-planar shape of the reflecting layers. For a hypothetical array of ridges with identical irregular layers, the simple grating theory would apply, and the result would be sharp diffraction peaks irrespective of either external or internal structure (See Supplementary Information, Fig. S2(h) for the calculated angular reflection of a hypothetical array of identical irregular units)^17^. Also, the arrays used in the calculations consisted of perfectly shaped units. Thus, the agreement between the calculated and experimental spectra indicates that the etch damage observed in Fig. 3b,c does not contribute significantly (More detailed information on the effect of etch damage can be found in Supplementary Information, Fig. S7).

### Coating application

**Figure 5:**
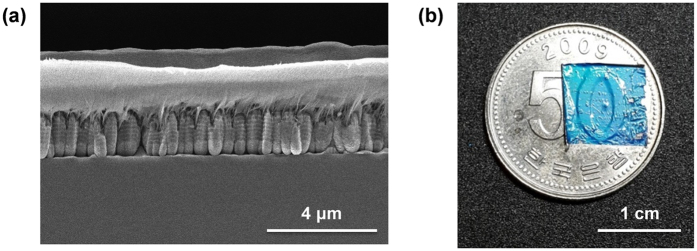
Parylene-deposited film. (**a**) Cross-sectional SEM image of a parylene deposited ridge structure with irregular layers. (**b**) The parylene deposited film attached on a Korean coin. The film follows the complex contours of the coin conformably.

In the present case, the overall film remains planar, with a total thickness of less than 2 μm, with features that are fully controlled via lithography and etching techniques. This greatly facilitates developing applications, as we here demonstrate by developing a robust coating application using parylene, a conformal protective polymer coating that is already in wide commercial use^37^, as a protective layer. Figure 5a shows a cross-section SEM image of an irregular-layered structure after parylene deposition. Full conformal coating and uniform infiltration of the inter-ridge space can be observed. After the deposition, the mechanical strength of the parylene layer enables detachment of the entire reflecting layer from the Si substrate, with the silica balls acting as the sacrificial layer. Due to the flexibility and thinness of the entire reflecting layer, conformal coating of arbitrary, non-planar shapes is possible. As shown in Fig. 5b, the film conformably coats the complex, convex pattern of a coin. The film naturally shows some specular reflection on the boundary of air and parylene, but retains the optical property of broad-angle blue reflection. (See Supplementary Information, Fig. S8 for the analysis of parylene coating). In fact, the film is robust enough to be dunked and folded in liquid nitrogen without suffering visible damage (see Supplementary Video).

## Conclusions

In conclusion, we have proposed and fabricated a structural color reflector that reproduces the structural hierarchy of disorder found in *Morpho* butterflies to generate bright, broad-angle blue color reflection. Calculations suggest, and experiments prove, that the shape of the reflecting ridges profoundly affect the color as well as the multilayer interference. Furthermore, an *inter-structural disorder* without positional disorder is demonstrated to be just as effective for broad-angle reflectance, paving the way for an alternative and possibly more viable generation of broad-angle reflection of *Morpho* blue.

## Methods

### Fabrication of Morpho-inspired structure

A monolayer of randomly sized silica microspheres with diameters ranging from 250 nm to 440 nm was spin-coated on a Si wafer, and then fixed to the Si substrate by a high temperature annealing (1000 °C) with O_2_ gas^34^. A 300 nm thick layer of Cr is then deposited on the silica microspheres to serve as the absorption layer, and 8 pairs SiO_2_/TiO_2_ layers of 95 nm and 45 nm, respectively, were deposited directionally using low-pressure sputter deposition. The directionality of the deposition process mostly preserves the random spatial variation of the silica ball substrate, and creates irregular layers with random variations in the vertical position of the layers. To form the ridges, a 100 nm thick layer of Cr was deposited on the multilayered films, followed by spin-coating of a 150 nm thick layer of photoresist (AZ-GXR601). A line pattern with a periodicity of 700 nm was then formed by exposing the photoresist to an interference pattern formed by a 457.9 nm Ar laser line followed by development (AZ-MIF300 developer). Using the line pattern as the oxidation barrier, the exposed Cr surface is oxidized by exposure to an O_2_ plasma. The photoresist is removed by dipping in acetone and DI water, and the non-oxidized Cr regions are selectively wet-etched using a Cr etchant (CE-905N). Finally, dry etching of the multilayer thin film in a CHF_3_ plasma (ICP-RIE: 100 W & 50 W, CHF_3_ (20 sccm), 15 mTorr, 2000 s), using the oxidized Cr surface as the etch barrier, is used to form the ridged structure.

### Simulation methods

The simulation for the structural modelling (Fig. 2) is performed using a two dimensional finite element method (COMSOL™) with perfectly matched layer (PML) boundary condition. A monochromatic plane wave incident normally on the film was used for incident light. Unless stated otherwise, all results are for unpolarised light, meaning that the intensities for TE- and TM-polarization are averaged. Far-field reflected intensities were obtained by near-to-far-field transformation. Each ridge is composed of 8 pairs of SiO_2_/TiO_2_ layers with experimentally determined refractive index values (see Supplementary Information, Fig. S9 for the values of refractive index). For the array structures, 23 multilayered ridges were calculated, and results from 10 statistically identical structures were averaged.

The simulation for the optical analysis of fabricated structures (Fig. 4) is performed with an in-house FEM solver described in more detail in ref. 20 with an updated boundary condition for incident waves as described in ref. 38. For a tapered ridge structure with regular layers, the overall reflection of 20 of the tapered units, obtained from SEM images, placed next to each other – each unit being 700 nm – was used. This corresponds to performing the following calculation:


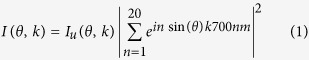


where *I*_*u*_ is the far-field intensity of a single unit, *θ* is the reflection angle, *k* is the wave vector, and *i* is the imaginary unit.

The simulation of a tapered ridge structure with irregular layers is the ensemble averaged response of 200 realizations of different random combinations of irregular unit structures selected from a pool of 100 unit responses. This means that the overall response was calculated as


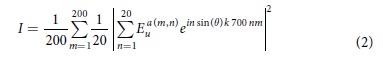


where *E*_*u*_^*a(m,n*)^ is the electrical far-field (scaled such that its square magnitude equals intensity) of the *a*th unit structure, where – for each (*n, m*) – *a* is drawn from a pool of uniform integers from 1 to 100. The dependent variables for *I* and *E* have been dropped for clarity. The basis for this approach is discussed in ref. 17 and the validity discussed in ref. 21. A verification of the approach for these specific structures is also provided as supporting material in the Supplementary Information, Fig. S10.

## Additional Information

**How to cite this article**: Song, B. *et al*. Reproducing the hierarchy of disorder for *Morpho*-inspired, broad-angle color reflection. *Sci. Rep.* **7**, 46023; doi: 10.1038/srep46023 (2017).

**Publisher's note:** Springer Nature remains neutral with regard to jurisdictional claims in published maps and institutional affiliations.

## Supplementary Material

Supplementary Information

Supplementary Video
