# Supplementary material for: Reproducing the hierarchy of disorder for Morpho-inspired, broad-angle color reflection
Source: Sci Rep. 2017 Apr 7;7:46023. doi: 10.1038/srep46023 (PMC5384085; doi:10.1038/srep46023)
Supplement: Supplementary Information [file srep46023-s1.pdf]

Supplementary Information for

Reproducing the hierarchy of disorder for *Morpho*-inspired, broad-angle color reflection

Bokwang Song[1], Villads Egede Johansen[2,3], Ole Sigmund[3] and Jung H. Shin[4,1,]*

[1]*Dept. of Physics, KAIST, 335 Gwahangno, Yuseong-Gu, Daejeon, Rep. of Korea*

[2]*Department of Chemistry, University of Cambridge, Lensfield Road, CB2 1EW, Cambridge, United Kingdom*

[3]*Department of Mechanical Engineering, Solid Mechanics, Technical University of Denmark, Bld. 404, DK-2800 Kgs. Lyngby, Denmark*

[4]*Graduate School of Nanoscience and Technology KAIST, 335 Gwahangno, Yuseong-Gu, Daejeon, Rep. of Korea*

Correspondence and requests for materials should be addressed to B.S. (email: bokwang@kaist.ac.kr)

Figure S1

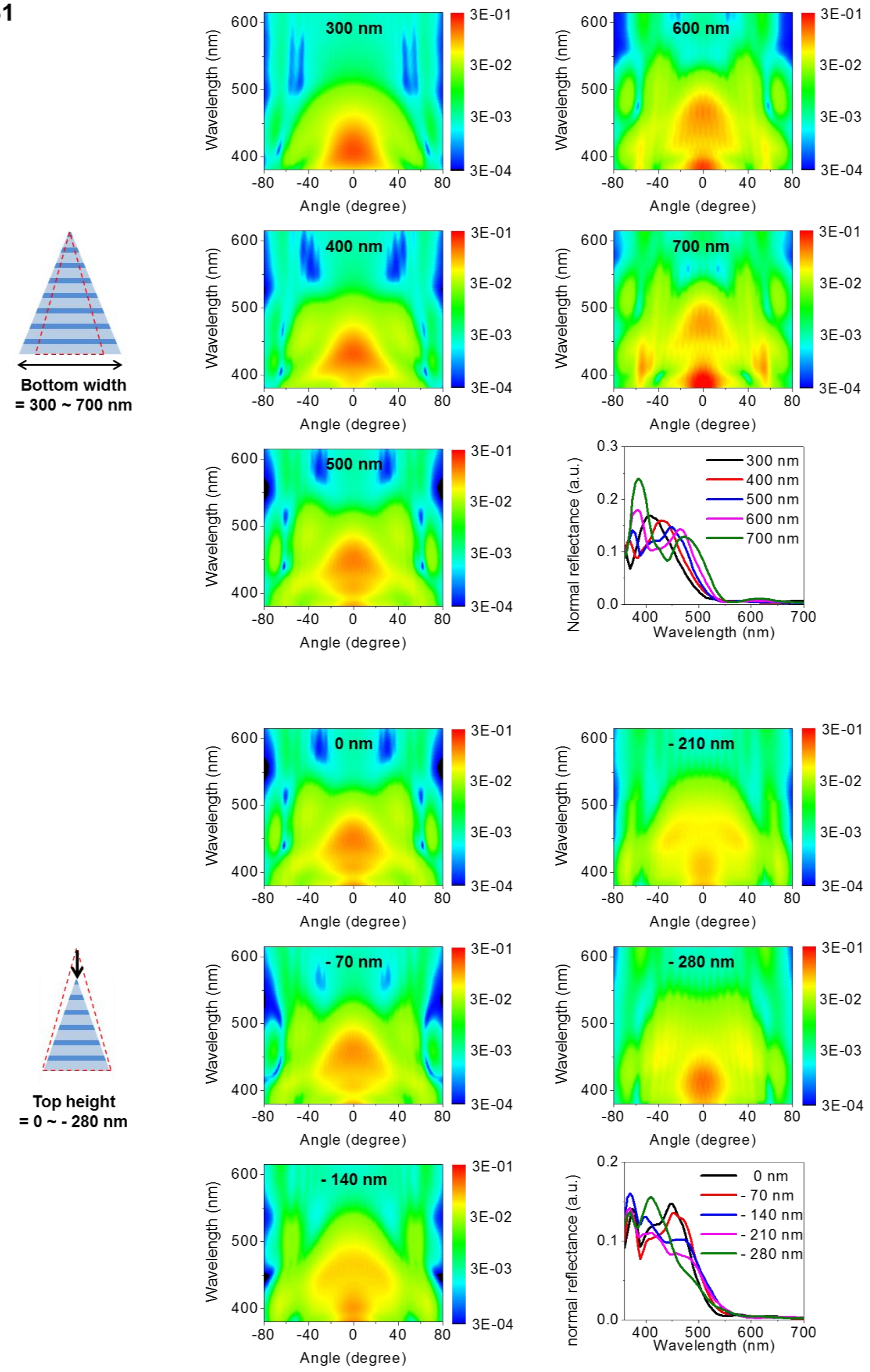

Bottom width
= 300 ~ 700 nm
300 nm
400 nm
500 nm
600 nm
700 nm
Wavelength (nm)
Angle (degree)
3E-01
3E-02
3E-03
3E-04
Normal reflectance (a.u.)
300 nm
400 nm
500 nm
600 nm
700 nm
Top height
= 0 ~ - 280 nm
0 nm
- 70 nm
- 140 nm
- 210 nm
- 280 nm
normal reflectance (a.u.)
0 nm
- 70 nm
- 140 nm
- 210 nm
- 280 nm

Figure S1. Effect of the ridge shape on the unit response.

The effect of the ridge shape was investigated using two dimensional finite element method (COMSOL™) with perfectly matched layer (PML) boundary conditions. Far-field reflected intensities were obtained by near-to-far-field transformation. Each ridge is composed of 8 pairs of SiO_2/TiO_2 layers with experimentally obtained refractive index values. Two variables (the bottom width of the ridge for a fixed height and the height for a fixed width) were varied, and their effect on the reflection spectrum upon normal incidence investigated.

We find that while all show suppression of reflection in the red, the details of the reflection spectra depend on the detailed shape of the ridge even though the multilayer period and the multilayer materials were held constant throughout the simulation. This demonstrates that the shape of the reflecting multilayer ridge contributes significantly to the final color of the *Morpho* butterflies. Indeed, the tapered shape is quite effective in generating pure blue.

Figure S2

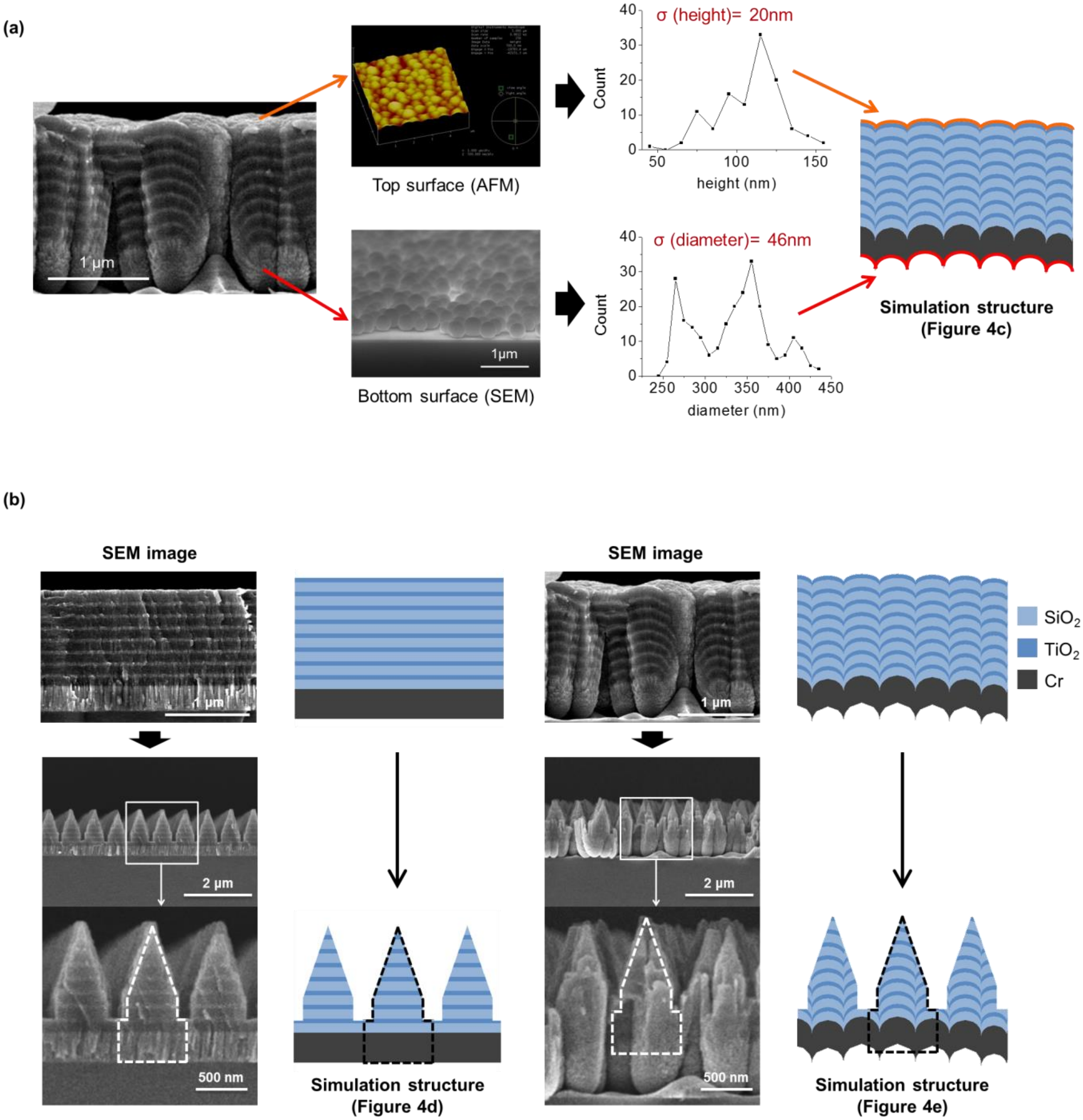
(a)
1 μm
Top surface (AFM)
Bottom surface (SEM)
1μm
σ (height)= 20nm
Count
height (nm)
σ (diameter)= 46nm
diameter (nm)
Simulation structure
(Figure 4c)
(b)
SEM image
1 μm
2 μm
500 nm
Simulation structure
(Figure 4d)
Simulation structure
(Figure 4e)
SiO2
TiO2
Cr

Figure S2

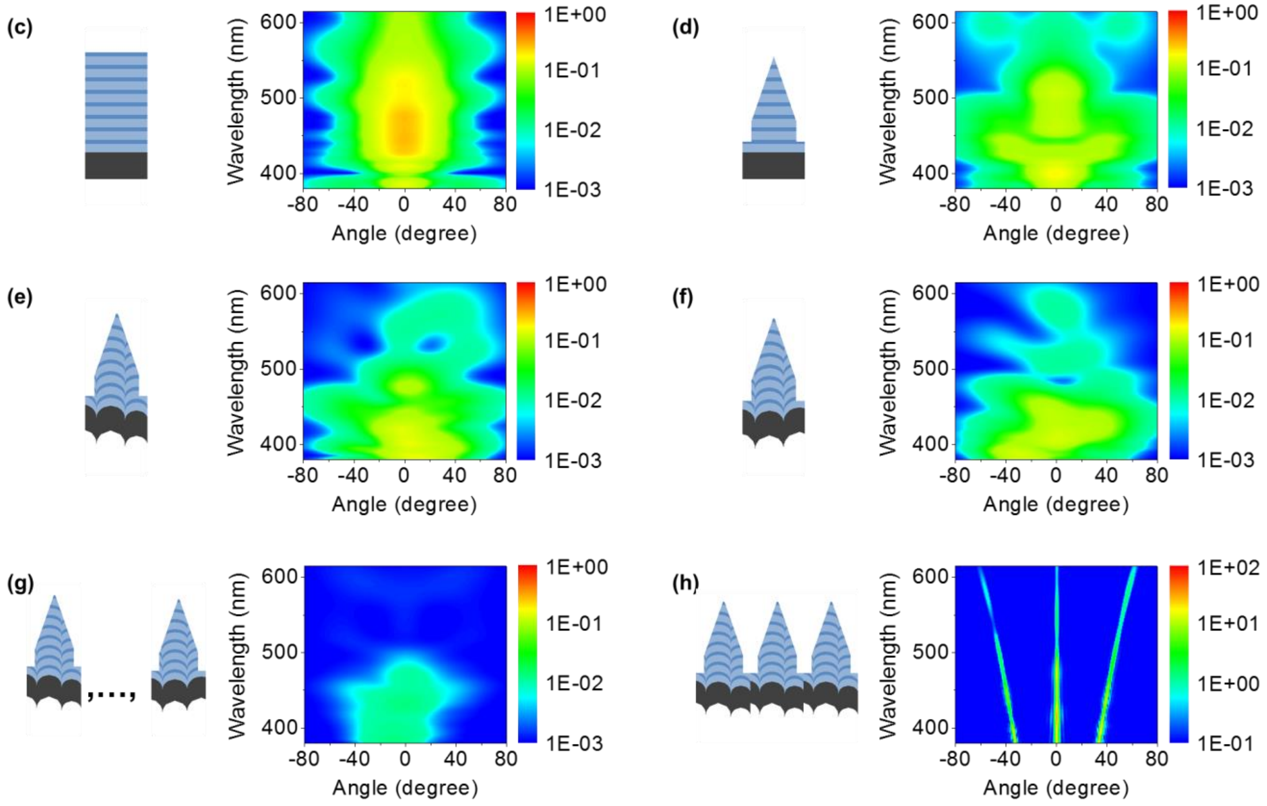

Figure S2. Simulation structures and results. (a) The irregular shape of layers measured by AFM and SEM. The standard deviation of vertical disorder is reduced from 46 nm to 20 nm after deposition of the multilayer. **(b)** The actual shape of the ridges obtained from SEM images. Calculated unit responses of **(c)** a rectangular ridge with regular layers, **(d)** a tapered ridge with regular layers, and **(e,f)** two different tapered ridges with irregular layers. **(g)** Calculated unit response averaged over the complex far-fields of 30 randomly generated tapered ridges with irregular layers. Averaging unit intensities, one at a time, clearly gives a wrong result as compared to Figure 4(f). The ensemble average approach in Equation (2) is therefore needed to represent the scattering response. **(h)** Calculated angular reflection of a hypothetical array of identical irregular units.

Figure S3

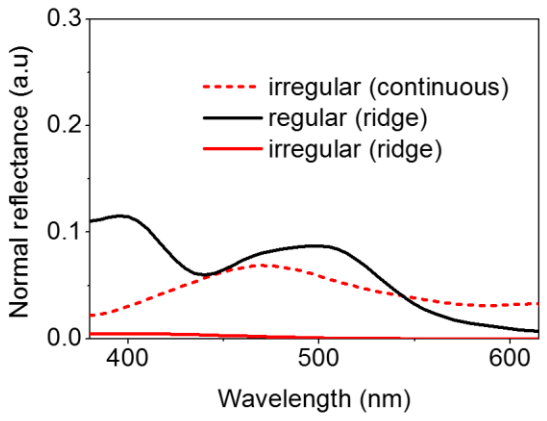

Figure S3. The calculated reflection spectra of the fabricated structures under normal incident light conditions (the same data as in Fig. 4d-f). Due to the finite detector size, the scattered light from the irregular structure (also reflecting around the non-specular angle) can be collected by the detector, while there is ideally no scattering around the specular angle from the regular structure. For this reason, the calculated normal reflection of the irregular structure (Fig. S3) is relatively lower than the measured one (Fig. 4a).

Figure S4

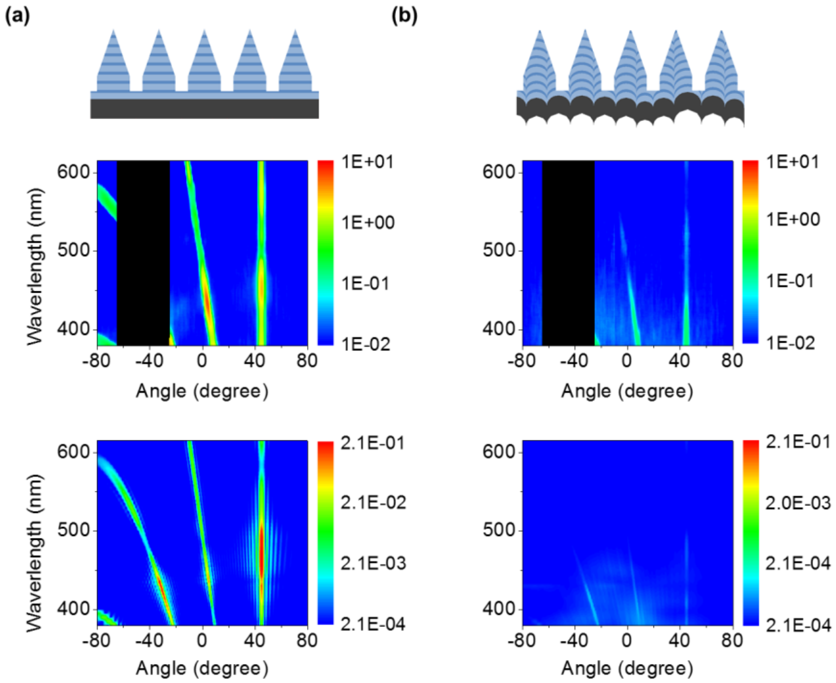
(a)
(b)
Wavelength (nm)
600
500
400
-80
-40
0
40
80
Angle (degree)
1E+01
1E+00
1E-01
1E-02
2.1E-01
2.1E-02
2.1E-03
2.1E-04
2.0E-03

Figure S4

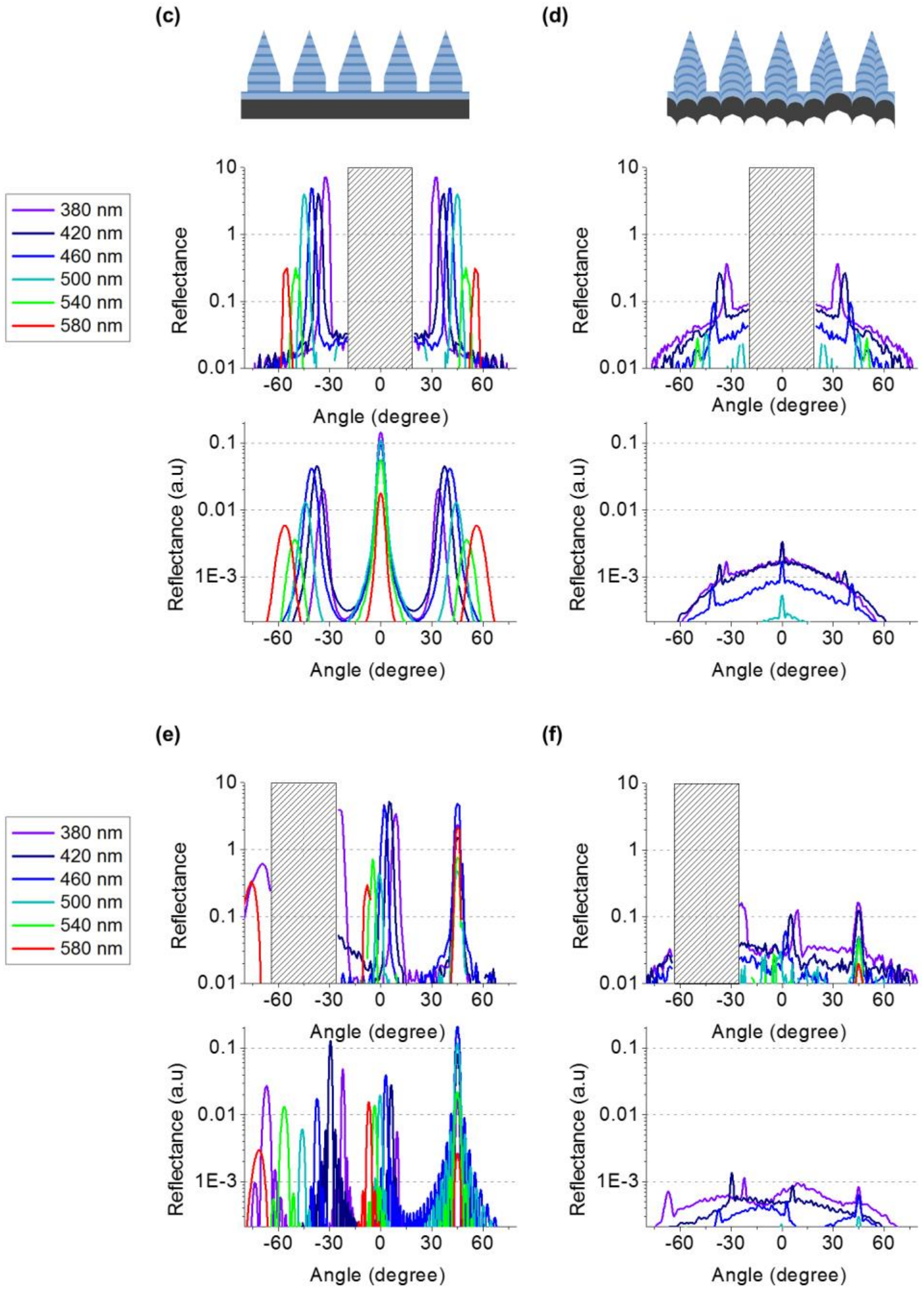

Figure S4. (a,b) Experimentally measured (middle) and calculated (bottom) reflection spectra of the fabricated structures for a 45° incident angle. A schematic of the corresponding structure is given on top. **(c,d)** Angle-resolved spectra of selected wavelengths from 380 to 580 nm with 40 nm interval

under normal incident light conditions. A schematic of the corresponding structure is given on top. **(e,f)** Corresponding data for a 45° incident angle. Note that all graphs are plotted in log scale. All simulations were performed by the in-house FEM solver which was also used for the simulation of Fig. 4.

Figure S5

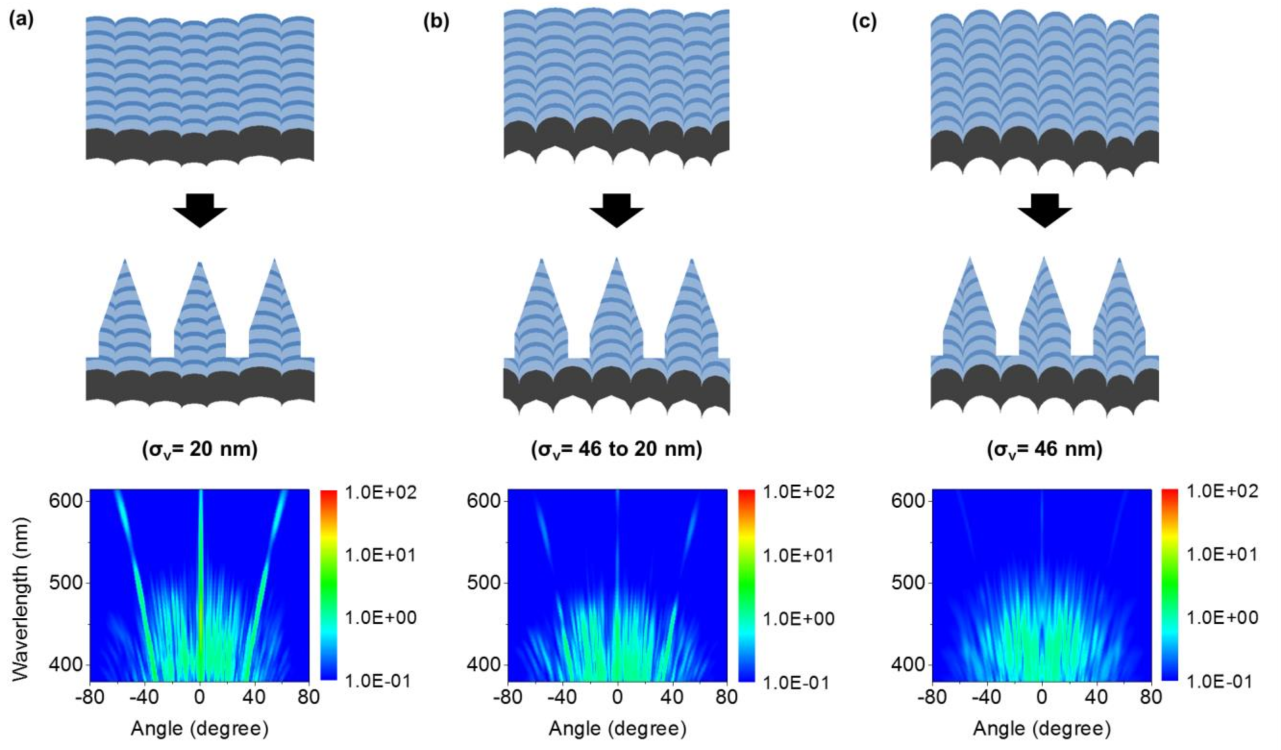

Figure S5. The effect of directionality of deposition.

The effect of directionality of deposition was investigated by the finite element method (COMSOL™) which was used for the simulation of Fig. 2. **(a)** Calculated far-field reflected intensities of a ridged structure with irregular layers. The standard deviation of vertical disorder remains 20 nm for all layers, indicating that the directionality of deposition is perfect. **(b)** Corresponding data for a ridged structure with irregular layers, whose vertical disorder linearly decreases from bottom to top layer, 46 nm to 20 nm, indicating that the directionality of deposition is not as perfect as the fabricated ridge structure. **(c)** Corresponding data for a ridged structure with irregular layers, whose vertical disorder is 46 nm for all layers, indicating that the directionality of deposition is perfect.

We find that increasing the directionality deposition is necessary for generating enough disorder. And increasing disorder of the original bottom substrate may be another solution.

Figure S6

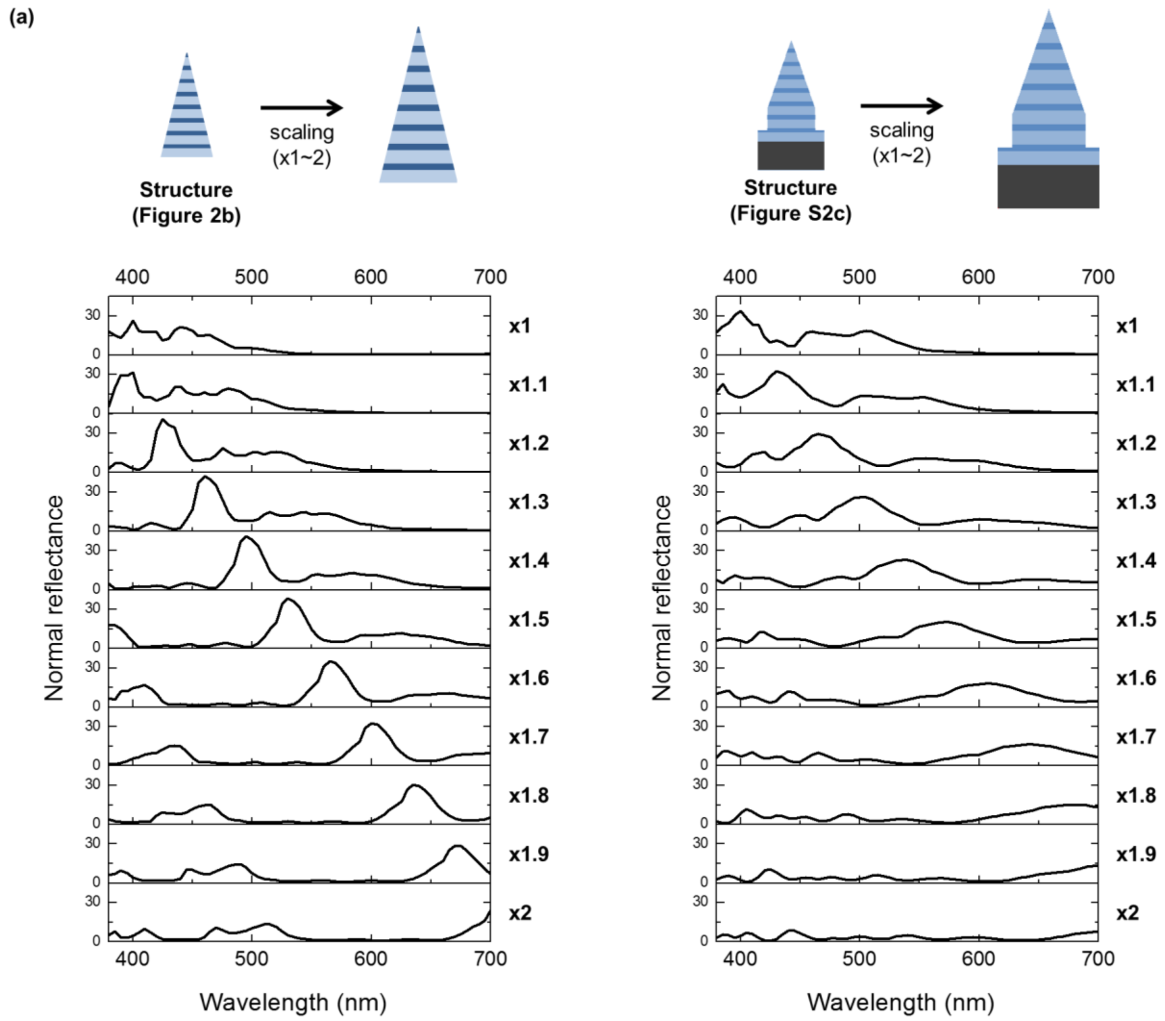

(a)
Structure
(Figure 2b)
scaling
(x1~2)
Structure
(Figure S2c)
scaling
(x1~2)
Normal reflectance
Wavelength (nm)
400
500
600
700
30
0
x1
x1.1
x1.2
x1.3
x1.4
x1.5
x1.6
x1.7
x1.8
x1.9
x2

Figure S6

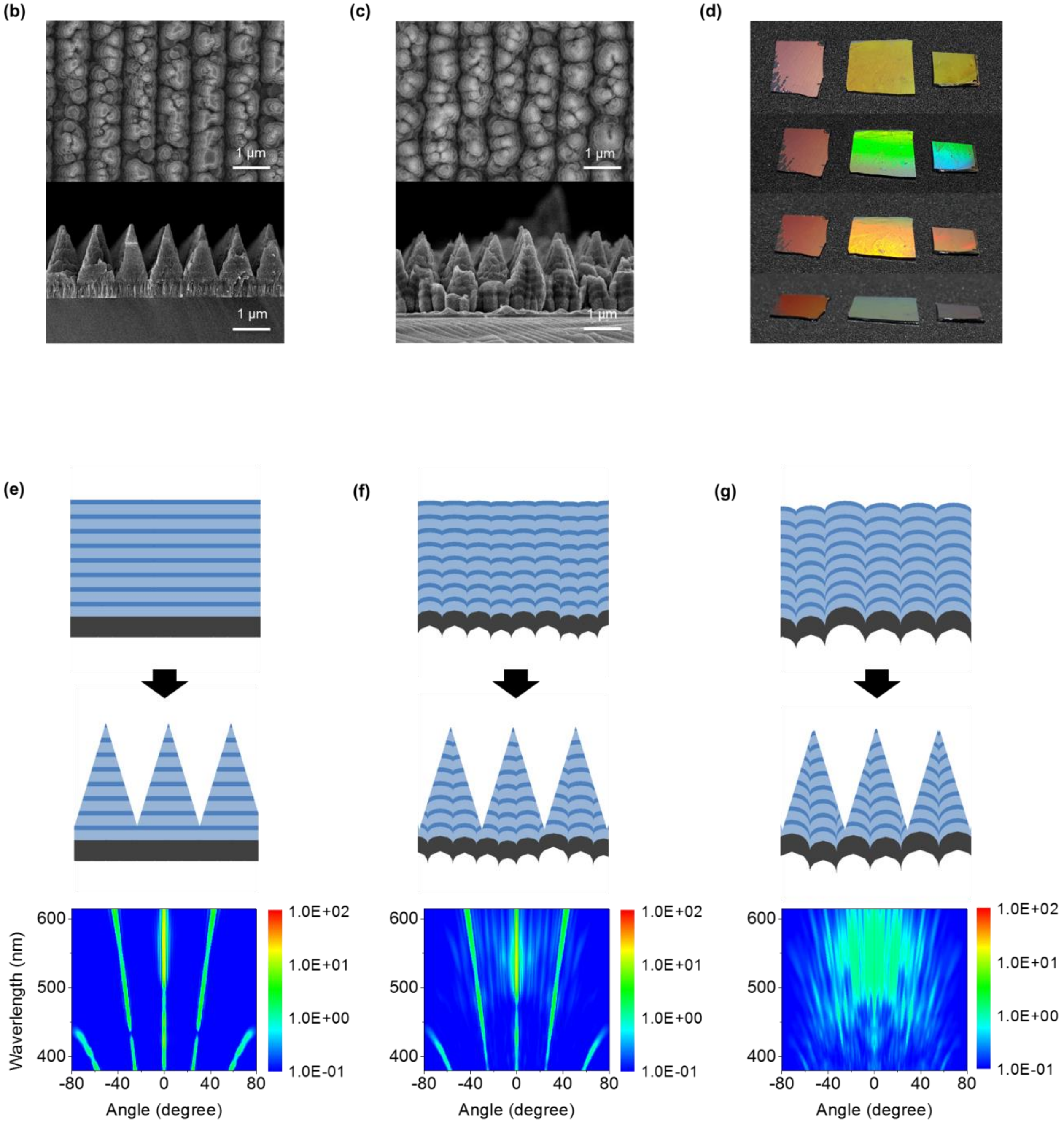
(b)
1 µm
1 µm
(c)
1 µm
1 µm
(d)
(e)
(f)
(g)
Wavelength (nm)
600
500
400
-80 -40 0 40 80
Angle (degree)
1.0E+02
1.0E+01
1.0E+00
1.0E-01

Figure S6. Generating red color by scaling the system.

(a) Normal reflectances (absolute values) of the ridge structures as a result of upscaling.

(b) SEM images of the regular multilayer after ridge formation. The layer thicknesses of SiO_2 and TiO_2 are 142 nm and 68 nm (1.5 times the blue-structure), respectively, and the periodicity of lithography is 900 nm. **(c)** Corresponding data for the irregular multilayer after ridge formation. The microspheres used for the blue-structure were coated for the disordered substrate. **(d)** Optical images of the fabricated films under normal illumination. From left to right: a continuous structure with irregular layers, a ridge structure with regular layers (Fig. S6b), and a ridge structure with irregular layers (Fig. S6c). The viewing angles are, from the top, approximately 10, 35, 45, and 55 degrees. The red-colored structure also shows a blueshift of its hue just as seen for the blue structure. The ridged structures show a yellowish color, while the continuous structure shows red color. **(e)** Calculated far-field reflected intensities of a ridged structure with regular layers. The simulation was performed by finite element method (COMSOL™) using the actual shape of the ridges obtained from SEM images (Fig. S6b). **(f)** Corresponding data for a ridged structure with irregular layers, whose structural parameters are obtained by SEM images (Fig. S6c). **(g)** Corresponding data for a ridged structure with irregular layers, assuming that 1.5 times the size of microspheres is used for the disordered substrate.

We find that the red-shifted color can be defined simply by upscaling the multilayer thickness. However, for broad-angle reflection of the red-shifted color, it is obvious that the scale of disorder has to increase as multilayer thickness increases.

Figure S7

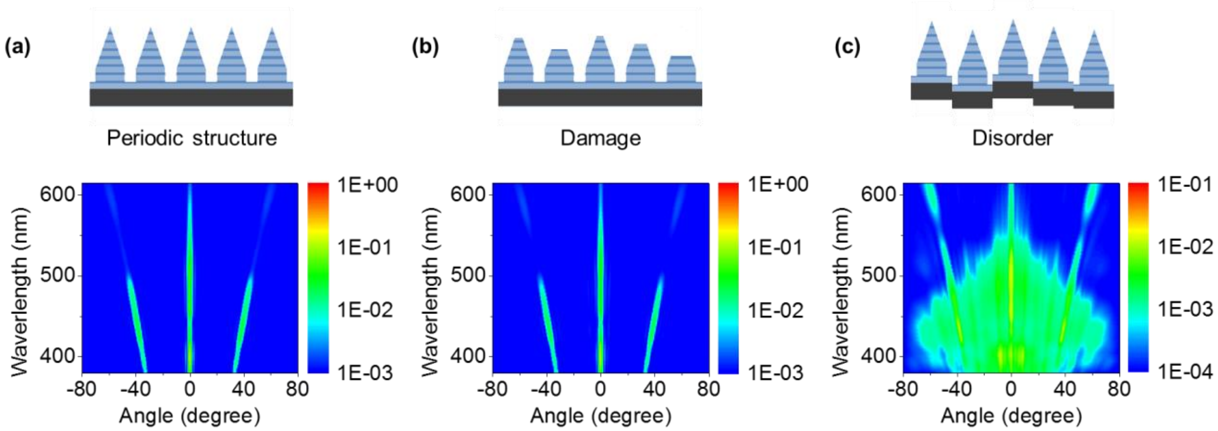

Figure S7. Comparison between the effect of disorder and damage by numerical simulation.

The effect of etch damage was investigated using two dimensional finite element method (in-house FEM, COMSOL) with perfectly matched layer (PML) boundary condition. 15 multilayered ridges were calculated, and each ridge was composed of 8 pairs of SiO_2/TiO_2 layers and a Cr layer with experimentally determined structural parameters and refractive index values.

(a) Calculated far-field reflected intensities of tapered multilayer grating. A schematic view of the structure is shown on top. **(b-c)** Calculated far-field reflected intensities of tapered multilayer grating with **(b)** damage (vertical damage: 0~140 nm with 10 nm interval, standard deviation: 45 nm), and **(c)** disorder (vertical offset: 0~140 nm with 10 nm interval, standard deviation: 45 nm). Results from 20 statistically identical structures were averaged.

By numerical simulation, we confirm that *inter-structural disorder* is quite effective for broad-angle reflection. On the other hand, both the strong specular reflection and the sharp diffraction peaks remain almost the same after etch damage, even though the standard deviation of damage is the same as that of the *inter-structural disorder*. We conclude that etch damage has little effect on the overall optical response of the system compared to disorder.

Figure S8

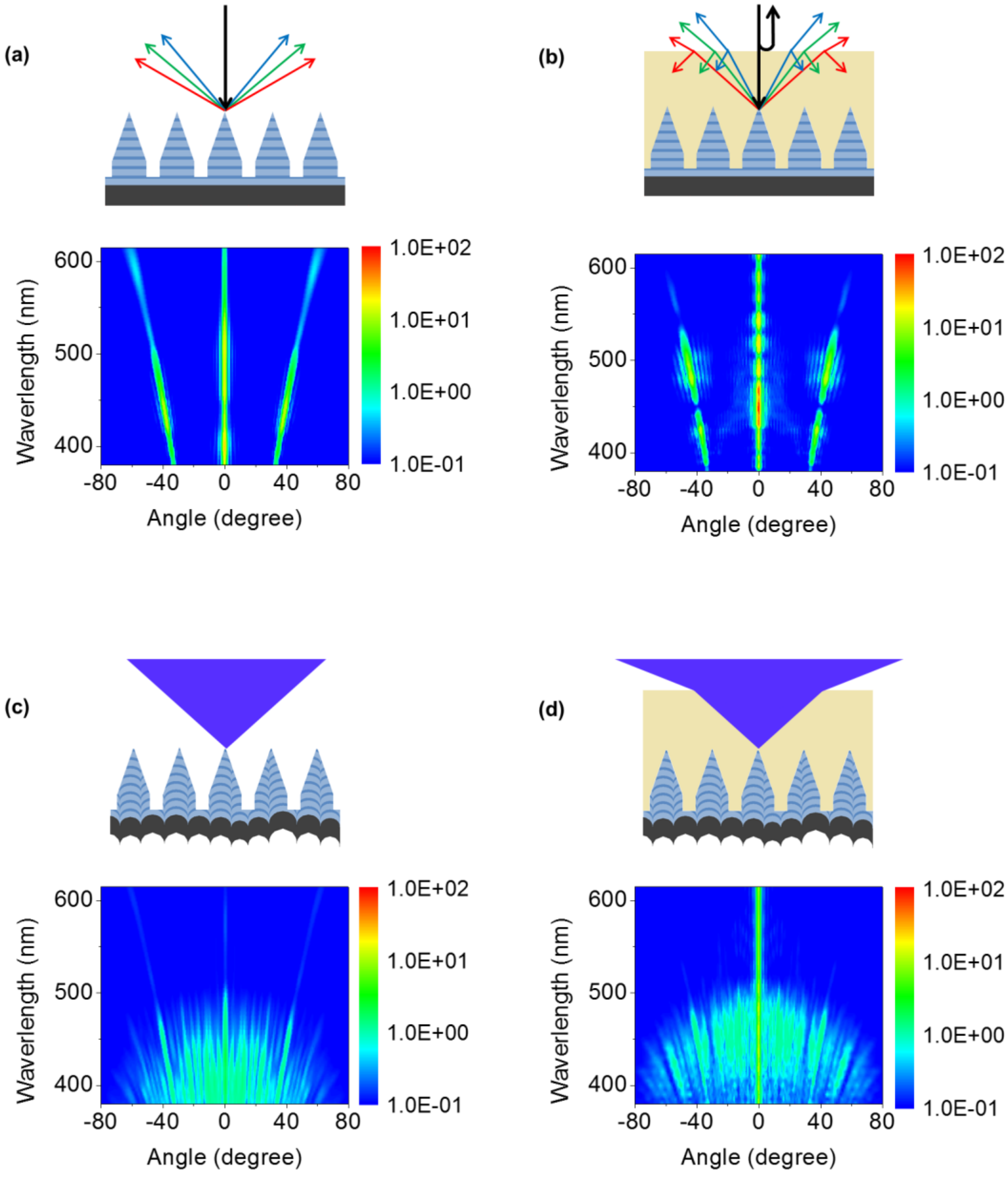

Figure S8. Effect of parylene coating.

The effect of parylene coating was investigated by the finite element method (COMSOL™) which was used for the simulation of Fig. 2. **(a)** Calculated far-field reflected intensities of a ridged structure with regular layers. **(b)** Corresponding data for a ridged structure with regular layers coated by parylene.

Refractive index of parylene is 1.639. Total thickness of film is 4 μm obtained from the SEM image of Fig. 5a. **(c)** Corresponding data for a ridged structure with irregular layers, whose vertical disorder is 46 nm to 20 nm from bottom to top layer. **(d)** Corresponding data for a ridged structure with irregular layers coated by parylene. A schematic of the corresponding structure together with reflection property is given on top.

By parylene coating, some enhancement of normal reflection is observed due to specular reflection on the boundary of air and parylene as indicated in the schematic of (b). But, two positive effects for broad-angle blue reflection are also confirmed. First, diffraction peaks of longer wavelength region are suppressed by internal reflection as indicated in a schematic of (b). Second, the reflection angle is slightly broadened due to refraction as indicated in a schematic of (d).

Figure S9

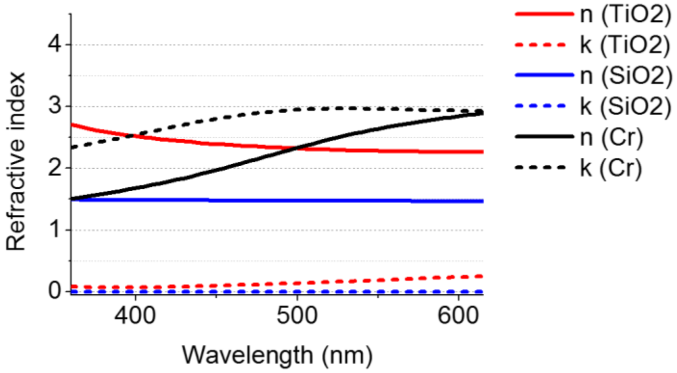

Figure S9. The refractive index values measured by ellipsometry.

Figure S10

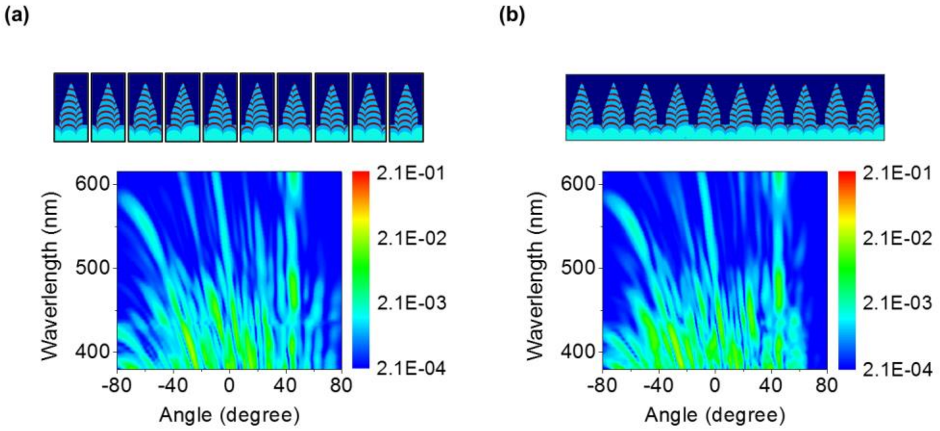

Figure S10. Verification of the approximate simulation. (a) 10 units are simulated individually, and their unpolarised far-field response for 45 degree incident light is found according to equation (2) with no averaging (m=1). **(b)** A simulation of the whole structure at once.

The two responses are similar with only minor differences. We conclude from this, that when averaging the incoherent response of 200 such structures, we get a good approximation of the result which could be obtained by full simulations of the same number of large simulation domains.

Supplementary Video

This video shows the parylene-deposited *Morpho*-inspired structure shown in Figure 5, being shaken in liquid nitrogen. The film is robust enough to be repeatedly folded in liquid nitrogen without suffering visible damage.
